# SOHSite: incorporating evolutionary information and physicochemical properties to identify protein *S*-sulfenylation sites

**DOI:** 10.1186/s12864-015-2299-1

**Published:** 2016-01-11

**Authors:** Van-Minh Bui, Shun-Long Weng, Cheng-Tsung Lu, Tzu-Hao Chang, Julia Tzu-Ya Weng, Tzong-Yi Lee

**Affiliations:** Department of Computer Science and Engineering, Yuan Ze University, Taoyuan, 320 Taiwan; Department of Obstetrics and Gynecology, Hsinchu Mackay Memorial Hospital, Hsin-Chu, 300 Taiwan; Mackay Junior College of Medicine, Nursing and Management, Taipei, 112 Taiwan; Department of Medicine, Mackay Medical College, New Taipei City, 252 Taiwan; Graduate Institute of Biomedical Informatics, Taipei Medical University, Taipei, 110 Taiwan; Innovation Center for Big Data and Digital Convergence, Yuan Ze University, Taoyuan, 320 Taiwan

**Keywords:** *S*-sulfenylation, Sulfenic acids, Support vector machine, Physicochemical properties

## Abstract

**Background:**

Protein *S*-sulfenylation is a type of post-translational modification (PTM) involving the covalent binding of a hydroxyl group to the thiol of a cysteine amino acid. Recent evidence has shown the importance of *S*-sulfenylation in various biological processes, including transcriptional regulation, apoptosis and cytokine signaling. Determining the specific sites of *S*-sulfenylation is fundamental to understanding the structures and functions of *S*-sulfenylated proteins. However, the current lack of reliable tools often limits researchers to use expensive and time-consuming laboratory techniques for the identification of *S*-sulfenylation sites. Thus, we were motivated to develop a bioinformatics method for investigating *S*-sulfenylation sites based on amino acid compositions and physicochemical properties.

**Results:**

In this work, physicochemical properties were utilized not only to identify *S*-sulfenylation sites from 1,096 experimentally verified *S*-sulfenylated proteins, but also to compare the effectiveness of prediction with other characteristics such as amino acid composition (AAC), amino acid pair composition (AAPC), solvent-accessible surface area (ASA), amino acid substitution matrix (BLOSUM62), position-specific scoring matrix (PSSM), and positional weighted matrix (PWM). Various prediction models were built using support vector machine (SVM) and evaluated by five-fold cross-validation. The model constructed from hybrid features, including PSSM and physicochemical properties, yielded the best performance with sensitivity, specificity, accuracy and MCC measurements of 0.746, 0.737, 0.738 and 0.337, respectively. The selected model also provided a promising accuracy (0.693) on an independent testing dataset. Additionally, we employed TwoSampleLogo to help discover the difference of amino acid composition among *S*-sulfenylation, *S*-glutathionylation and *S*-nitrosylation sites.

**Conclusion:**

This work proposed a computational method to explore informative features and functions for protein *S*-sulfenylation. Evaluation by five-fold cross validation indicated that the selected features were effective in the identification of *S*-sulfenylation sites. Moreover, the independent testing results demonstrated that the proposed method could provide a feasible means for conducting preliminary analyses of protein *S*-sulfenylation. We also anticipate that the uncovered differences in amino acid composition may facilitate future studies of the extensive crosstalk among *S*-sulfenylation, *S*-glutathionylation and *S*-nitrosylation.

**Electronic supplementary material:**

The online version of this article (doi:10.1186/s12864-015-2299-1) contains supplementary material, which is available to authorized users.

## Introduction

Post-translational modification (PTM) at the cysteine residues is essential to the dynamic functions of proteins. *S*-sulfenylation is a reversible covalent modification on the thiol group of cysteine residues by hydrogen peroxide, whereby the Cys-SH is oxidized to sulfenic (Cys-SOH), sulfinic (Cys–SO_2_H) and sulfonic acids (Cys–SO_3_H). More importantly, these changes contribute strongly to the regulation of protein function under both normal and oxidative stress conditions [1–4]. To date, a little more than 200 transcription factors, signaling proteins, metabolic enzymes, proteostasis regulators, and cytoskeletal components have been identified as S-sulfenylated proteins [5]. Thus, investigation of *S*-sulfenylated proteins is crucial to our understanding of the function and regulation of this oxidative post-translational modification at cysteine residues.

Several chemoproteomic approaches have been developed for identifying specific sites in proteins that undergo *S*-sulfenylation [6–10]. Nevertheless, these experimental methods are often expensive and time-consuming. Further, the lack of information about *S*-sulfenylation sites and the multiplicity of redox changes contribute to many false-positive identifications [11]. To facilitate the efficiency of experimental characterization and search for S-sulfenylated peptides, construction of a resource database is necessary, and possible with the development of powerful bioinformatics technologies. In spite of the availability of several published algorithms and public servers that aim to help analyze the reactive and oxidative sites [12, 13] of cysteine residues, there are no confirmed reports about the site-specific information of *S*-sulfenylated proteins, except for the chemical characterization of over 1000 *S*-sulfenylation sites on more than 700 proteins in cell culture experiments [11].

The present study (SOHSite) concentrated on the computational identification and characterization of *S*-sulfenylation sites. To discriminate between *S*-sulfenylation and non-*S*-sulfenylation sites, features including amino acid composition (AAC), amino acid pair composition (AAPC), position specific scoring matrix (PSSM), position weight matrix (PWM), amino acid substitution matrix (BLOSUM62), accessible surface area (ASA), and the physicochemical properties of proteins, were examined. Support vector machine [14] (SVM) was used to learn a predictive model from each feature, as well as hybrid combinations of the features. The SVM model with the best predictive performance would be selected based on the result of five-fold cross-validation. An independent testing set was applied to further evaluate the effectiveness of the chosen model. Finally, we investigated both key amino acid and hydrophobic attributes associated with *S*-sulfenylation sites.

## Materials and method

### Data collection and preprocessing

**Fig. 1:**
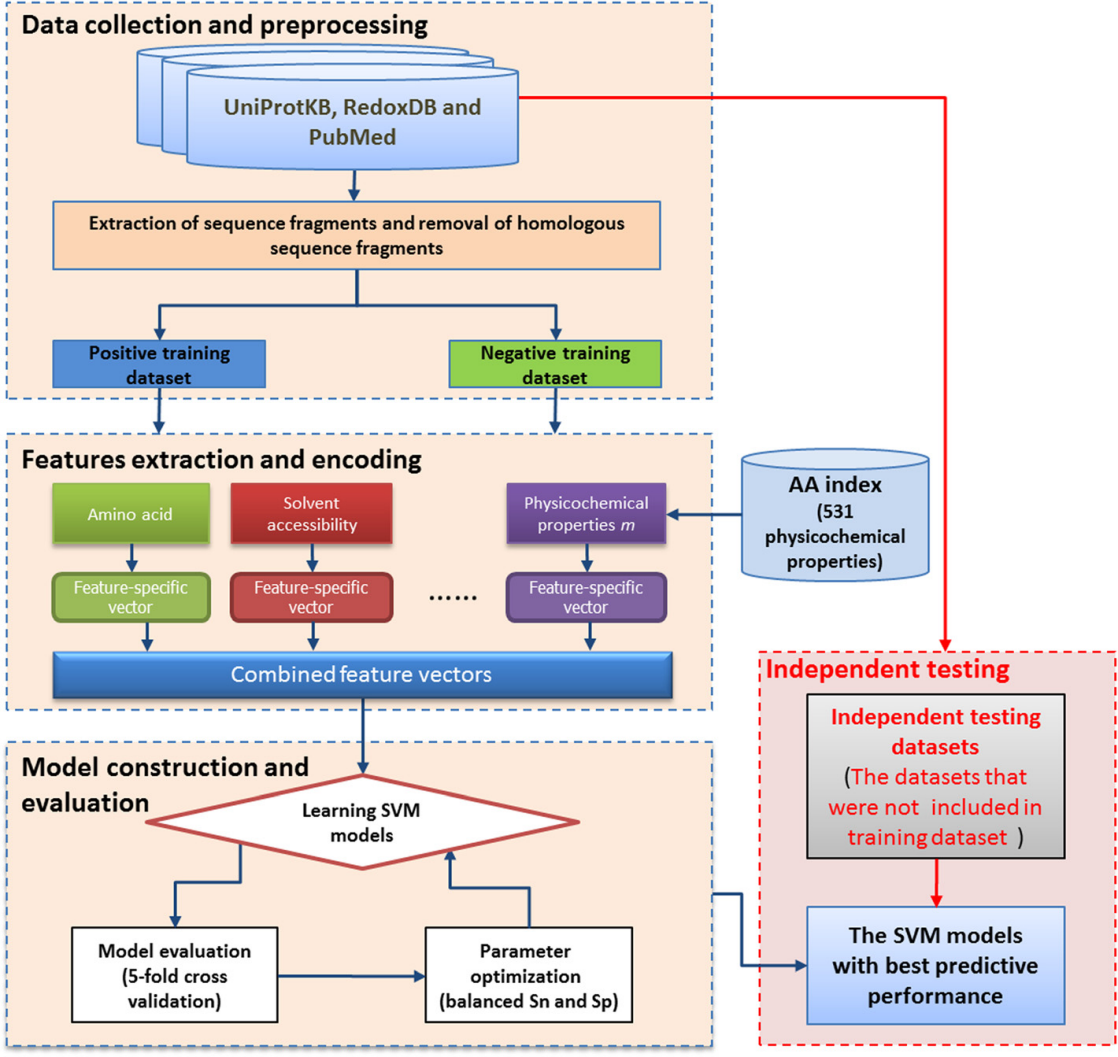
Analytical flowchart of SOHSite including data collection and preprocessing, features extraction and encoding, model construction and evaluation, and independent testing

Figure 1 presents the analytical flowchart of SOHSite. A majority of the experimental data used in this study was obtained from the Carroll laboratory database, which stores information on experimentally verified *S*-sulfenylated cysteines in humans based on the newly discovered *S*-sulfenyl-mediated redox regulation of the transcription factor H1F1A by SIRT6 [11]. As shown in Table 1, the data were composed of 1443 positive and 10521 negative data on 987 S-sulfenylated proteins. An additional set of data was collected from RedoxDB [12], UniProtKB [15] and other published literature. From the RedoxDB database, we obtained 102 S-sulfenylated sites (positive dataset) in 92 proteins, while the rest of the cysteine residues in these proteins were considered as non-*S*-sulfenylated cysteines (negative dataset). Moreover, properties characterized by X-Ray crystallography [16] were included, resulting in 33 *S*-sulfenylated and 143 non-*S*-sulfenylated sites. Finally, a dataset comprised of 17 *S*-sulfenylated and 97 non-*S*-sulfenylated cysteines was downloaded from UniProtKB. In order to generate the training dataset, the window length of 2*n* + 1 was used to extract sequence fragments centering at the *S*-sulfenylated or non-*S*-sulfenylated cysteines and containing *n* upstream, as well as *n* downstream, flanking amino acids. After extracting the sequence fragments with 2*n* + 1 window length (*n* = 10), we randomly categorized the dataset into independent testing set for the evaluation of real performance and training set for the construction of *S*-sulfenylated site prediction models.

**Table 1:** Data statistics of *S*-sulfenylated and non-*S*-sulfenylated sites

Data resource	*S*-sulfenylation sites (Positive data)	Non-*S*-sulfenylation sites (Negative data)
UniProtKB 201412	17	92
RedoxDB v1 (PMID: 22833525)	102	401
Yang J et al., 2014 [11] (PMID: 25175731)	1,443	10,521
Other Literature	33	143
Combined non-redundant dataset		
Training	1,145	8,368
Independent testing	289	2,108

To choose the best prediction model, a five-fold cross-validation approach was utilized to evaluate the performance of discriminating *S*-sulfenylated substrate sites from non-*S*-sulfenylated cysteine residues. Then, the model yielding the best prediction performance was further examined with an independent testing dataset. To prevent overestimation on performance evaluation, homologous sequences in the training and independent testing datasets were removed using 30 % sequence similarity as the filtering threshold, with reference to MASA [17]. As shown in (Additional file 1: Figure S1), two *S*-sulfenylated proteins with more than 30 % sequence identity were defined as homologous protein sequences. Then, two homologous sequences were specified to re-align the fragment sequences using a window length of 2*n* + 1, centered on the *S*-sulfenylated sites using BL2SEQ [18]. For two fragment sequences with 100 % identity, when the *S*-sulfenylated sites in the two proteins are in the same positions, only one site was kept while the other was discarded. Also, the training data had the higher priority so that if the sequence appeared in two datasets, the homologous fragment would be preserved in the training set and deducted from the testing set. The final positive and negative data consisted of 1145 and 8368 for the training dataset, and 289 and 2108 for the independent testing dataset, respectively.

### Features extraction and encoding

In this study, numerous sequence-based features, including amino acid composition (AAC), amino acid pair composition (AAPC), solvent-accessible surface area (ASA), amino acid substitution matrix (BLOSUM62), position-specific scoring matrix (PSSM), positional weighted matrix (PWM) and physicochemical properties (AAindex), were assessed to conduct the best prediction model. After the extraction of sequence fragments with a window size of 21-mer amino acids, each sequence was encoded based on the investigated features. The orthogonal binary coding mechanism is one of the most popular coding methods for transforming amino acids into numeric vectors, called 20-dimensional binary coding (20D) [19]. Each amino acid was represented by a vector with 20 letters. For example, alanine (A) would be encoded as “10000000000000000000” while cysteine (C) would be “01000000000000000000” and so on. For each sequence fragment, the length of feature vectors with a window size of 2*n* + 1 was set to (2*n* + 1) x 20 to represent the flanking amino acids surrounding the *S*-sulfenylation sites. Therefore, there were a total of k vectors {x_i_, *i* = 1, 2 …, *k*} corresponding to the number of *k* sequence fragments in the training and testing dataset. For a binary classification, the labels +1 and −1 were assigned to the positive and negative data, respectively.

For the representations of amino acid compositions around *S*-sulfenylation sites, the vectors x_i_ consisted of 21 elements for the amino acid composition (AAC) and 441 elements for the amino acid pair composition (AAPC). The 20 elements were defined as the occurrence frequencies of 20 amino acids in a sequence fragment, while the 400 elements were defined as the occurrence frequencies of 400 amino acid pairs in a sequence fragment. When the sequence fragments at the N- or C-terminus were less than 21-mer, non-existing residues were filled with “X” in the corresponding position. Therefore, a total of 21 types of amino acids and 441 types of amino acid pairs were presented in our setting. Additionally, the BLOcks Substitution Matrix (BLOSUM62) [20] was based on the alignments of amino acid sequences possessing no more than 62 % identity between two peptide sequences with 21 amino acids. With reference to the SulfoSite strategy [21], the position weighted matrix (PWM) of amino acids was determined using the non-homologous training data. The PWM described the frequency of occurrence of amino acids surrounding the *S*-sulfenylation sites, and was utilized to encode the sequence fragments. Each residue in the training dataset was represented by a matrix of *m* × *w* elements, where the window size of 21 was designated by *w* and the 21 elements including the 20 types of amino acids as well as the terminal signal was denoted by *m*. Position Specific Scoring Matrix (PSSM) profiles were generated from PSI-BLAST [22] against non-redundant sequences of *S*-sulfenylation sites. The scores were computed based on the multiple sequence alignment of proteins which may have similar structures with different amino acid compositions. In a PSSM profile, the rows in the matrix of (2*n* + 1) × 20 elements were centered on the substrate site, with 2*n* + 1 representing the window size and the number 20 denoting the position specific scores for each amino acid.

The solvent-accessible surface area (ASA), a feature representing the accessibility of an amino acid side-chain on the surface of a protein that experience post-translational modification [23], was also included as an attribute for the identification of S-sulfenylation sites. The RVP-Net [24, 25] was applied to compute the ASA value from the protein sequence due to the lack of experimentally verified tertiary structures of S-sulfenylated protein in the Protein Data Bank (PDB) [26]. Based on the information regarding the neighborhood amino acid composition, RVP-Net could predict the real ASA of a residue by using a neutral network approach. The possible mean absolute error, which was defined as the absolute difference between the predicted and experimental values of relative ASA per residue [25], was 18.0–19.5 %, for each measurement. The value of ASA represented the percentage of the solvent-accessible area of each amino acid on the protein. To compute the ASA values of all of the residues, full-length protein sequences were input into the RVP-Net. Then, the ASA values of amino acids surrounding the *S*-sulfenylation sites were extracted and normalized based on a scale from zero to one. In the investigation herein of secondary structure surrounding the SOH sites, additionally, PSIPRED [27] is employed to compute the secondary structure from the protein sequence. PSIPRED is a simple and reliable method for predicting secondary structure, which incorporates two feed-forward neural networks to analyze the output obtained from PSI-BLAST (Position Specific Iterated - BLAST) [22]. The output of PSIPRED is given in terms of “H,” “E” and “C” which stand for helix, sheet and coil, respectively.

The AAindex [28] (Version 9.1) contains a total of 544 amino acid indices which specify the physicochemical properties of twenty amino acids. After the amino acid indices with the value “NA” were eliminated, the physicochemical properties of the remaining 531 were examined to determine their ability to distinguish *S*-sulfenylated sites from the non-*S*-sulfenylated sites. A set of 20 numerical values corresponding to the physicochemical property of each of the 20 amino acids was specified. The sequence fragments were transformed from AAs surrounding S-sulfenylation sites into values associated with their physicochemical properties. In order to identify the significant physicochemical properties, the F-score method [29–33] has been applied to calculate a statistical value for each position surrounding *S*-sulfenylation sites. The F-score of the *i*th physicochemical feature is defined as:

1 $$ \mathrm{F}\hbox{-} \mathrm{score}\ (i) = \frac{{\left({{\overline{x}}_i}^{\left(+\right)}-{\overline{x}}_i\right)}^2+{\left({{\overline{x}}_i}^{\left(-\right)}-{\overline{x}}_i\right)}^2}{\frac{1}{n^{+}-1}{\displaystyle \sum_{k=1}^{n^{+}}{\left({x}_{k,i}^{\left(+\right)}-{{\overline{x}}_i}^{\left(+\right)}\right)}^2}+\frac{1}{n^{-}-1}{\displaystyle \sum_{k=1}^{n^{-}}{\left({x}_{k,i}^{\left(-\right)}-{{\overline{x}}_i}^{\left(-\right)}\right)}^2}} $$

where ${\overline{x}}_i$, ${{\overline{x}}_i}^{\left(+\right)}$ and ${{\overline{x}}_i}^{\left(-\right)}$ denote the average value of the *i*th feature in the whole, positive, and negative data sets, respectively; *n*^+^ denotes the number of positive data set and *n*^−^ denotes the number of negative data set; *x*_*k*,*i*_^(+)^ denotes the *i*th feature of the *k*th positive instance, and *x*_*k*,*i*_^(−)^ denotes the *i*th feature of the *k*th negative instance [34]. The prediction performances obtained from using the physicochemical properties individually were evaluated, and the properties were subsequently sorted in descending order based on the accuracy of prediction.

For the construction of predictive models, hybrid features were generated by combining two or more single features. In an attempt to obtain the highest predictive accuracy, the single features were selected based on their predictive performance. Prior to classification, the data needed to be scaled in the range of [−1, 1] to enhance the effectiveness of the results [35].

### Model training and evaluation

The training data set was used for building prediction models with the support vector machine (SVM). This binary classification utilizes a kernel function to transform the input samples into a higher dimensional space and attempts to find a hyper-plane to discriminate the two classes with maximal margin and minimal error. In our study, a public SVM tool (LIBSVM) [14] was implemented to build models that could discriminate between *S*-sulfenylation and non-*S*-sulfenylation sites. The radial basis function (RBF) $K\left({S}_i,{S}_j\right)={e}^{\left(-\gamma {\left\Vert s\right.}_i-{s}_j\left\Vert {}^2\right.\right)}$ was adopted as the kernel function. Two factors were included to enhance the performance: the RBF kernel was determined by the gamma parameter, while the softness of the hyper-plane was modulated by the cost parameter.

To choose the best final model, five-fold cross-validation was carried out for each feature to evaluate the predictive performance. The training dataset was divided into five subgroups with approximately equal size. The ratio of the testing set to the training set was 1:4 and the cross-validation process was repeated five times. The five validation results were then combined to generate a single estimation. Obviously, one of the benefits of k-fold cross-validation is the improvement on the reliability of evaluation because all of the original data, including the training and testing data sets, were considered and each subset should be tested only once [36] . To estimate the predictive performance of each trained model, measures such as sensitivity (Sn), specificity (Sp), accuracy (Acc) and Matthews Correlation Coefficient (MCC) were used:

2 $$ Sn=\frac{TP}{TP+FN} $$

3 $$ Sp=\frac{TN}{TN+FP} $$

4 $$ Acc=\frac{TP=TN}{TP=FP=TN=FN} $$

5 $$ MCC=\frac{\left(TP*TN\right)-\left(FN*FP\right)}{\sqrt{\left(TP+FN\right)}*\left(TN+FP\right)*\left(TP+FP\right)*\left(TN+FN\right)} $$

where TP, TN, FP and FN represented the number of true positives, true negatives, false positives and false negatives, respectively. The MCC reflects both the sensitivity (true positive rate) and specificity (true negative rate) of a predictive model. Sometimes, accuracy is not useful when the two classes are of very different sizes [37]. Therefore, the MCC is usually regarded as a balanced measure even if the two classes are of very different sizes. Finally, after selecting the best model with the highest MCC value, an independent testing was carried out to evaluate its real predictive power.

## Results and discussion

### Investigation of amino acid composition of S-sulfenylation site

**Fig. 2:**
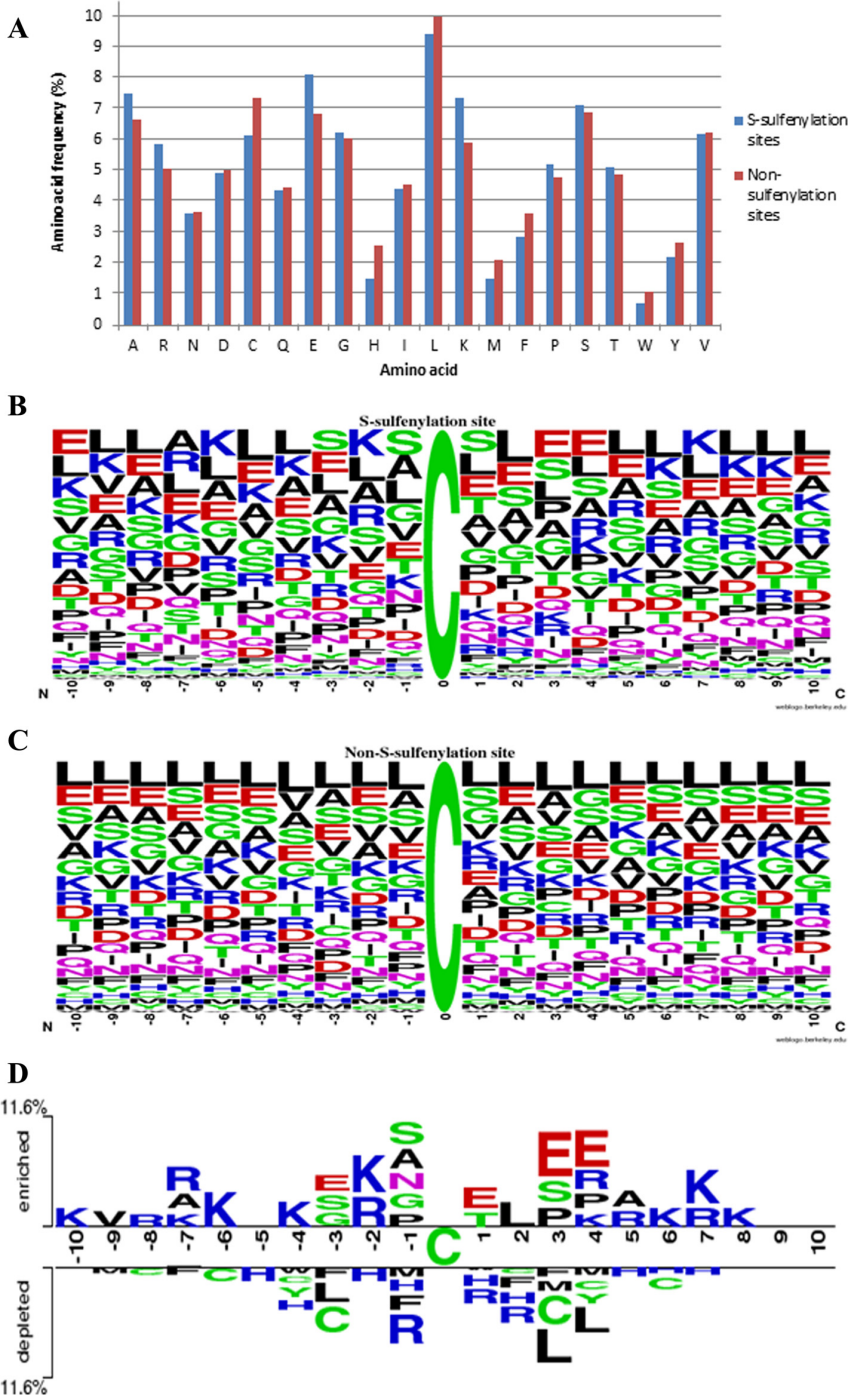
Amino acid composition of protein *S*-sulfenylation sites. **a** Comparison of amino acid composition between *S*-sulfenylation sites (*blue*) and non- *S*-sulfenylation sites (*red*). **b** Position-specific amino acid composition of *S*-sulfenylation sites. **c** Position-specific amino acid composition of non-*S*-sulfenylation sites. **d** TwoSampleLogo between *S*-sulfenylation sites (*positive data*) and non-*S*-sulfenylation sites (*negative data*)

Through sequence-based investigation, the frequency of twenty amino acids surrounding the *S*-sulfenylation sites revealed the amino acid composition that forms the substrate environment for protein *S*-sulfenylation. Figure 2A indicates that, at *S*-sulfenylation sites, A (Alanine), R (Arginine), E (Glutamic acid), and K (Lysine) residues occur at a higher frequency, while C (Cysteine) and H (Histidine) residues have a lower frequency of occurrence. In this investigation, WebLogo [38] was utilized to compute the position-specific amino acid composition for both *S*-sulfenylation (Fig. 2B) and non-*S*-sulfenylation (Fig. 2C). However, it is difficult to compare the amino acid composition between *S*-sulfenylation and non-*S*-sulfenylation sites at a specific position. To effectively identify *S*-sulfenylation sites, this study concentrated on notable differences between *S*-sulfenylated and non-*S*-sulfenylated sites. TwoSampleLogo [39] was employed to detect statistically significant differences in position-specific amino acid composition between the positive and negative datasets. Comparing between the 1145 positive data and 8368 negative data (Fig. 2D), it is obvious that three of the four aforementioned amino acids (R, E and K) play an important role in the flanking region of *S*-sulfenylation sites. In particular, the positively charged Lysine (K) and Arginine (R) residues had the highest ratio at positions −10, −8 ~ −6, −4, −2, and +4 ~ +8 (*p* < 0.01). In contrast, at positions −1, +1 and +2 that were close to the *S*-sulfenylation sites, a lack of positively charged residues was observed, while a noticeable abundance of negatively charged resides (Glutamic acid or E) was apparent at positions −3, +1, +3, +4. Interestingly, three polar residues, serine (S), asparagine (N), and glycine (G), appeared to have a higher frequency of occurrence compared to the rest of the amino acids at position −1. For non-*S*-sulfenylated sites, however, there was an abundance of neutral amino acids, including leucine (L), cysteine (C), histidine (H), methionine (M), phenylalanine (F) and tyrosine (Y), at positions ranging from −9 to +7, while arginine (R) residue seemed to be concentrated at three positions (−1, 1 and 2) around non-*S*-sulfenylation sites. Another evident difference is the amino acid composition surrounding S-sulfenylation sites at positions −6, −2, −1, +3 and +4. The analytical results indicated that the positions of amino acids relative to one another in the sequence play a vital role in discriminating between *S*-sulfenylation and non-*S*-sulfenylation sites.

**Fig. 3:**
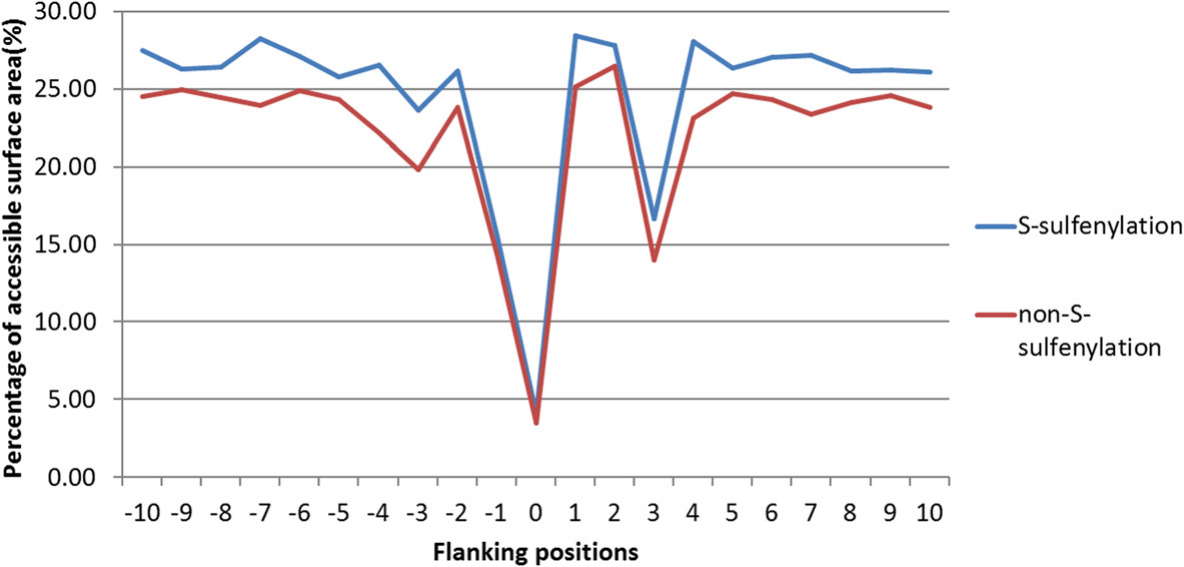
Comparison of the solvent-accessible surface area between *S*-sulfenylation and non-*S*-sulfenylation sites

Computed by the RVP-Net tool, ASA was also adopted as an attribute for the identification of S-sulfenylation sites. To discover how amino acids flanking the S-sulfenylation and non-S-sulfenylation sites may differ in their interaction with solvents, a comparison was performed using the average proportion of ASA in the 21-mer window (−10 ~ +10) as illustrated in Fig. 3. Amino acids surrounding the *S*-sulfenylated sites exhibit higher ASA compared to those around non-S-sulfenylation sites. A strong evidence for hydrophilicity at the S-sulfenylated substrate sites was found because the average percentage of ASA values of the flanking residues was higher than non-S-sulfenylated cysteines. Thus, hydrophilic amino acids flanking cysteine residues may determine their modification by sulfenylation.

### Performance evaluation of the trained models

Choosing suitable features is one of the crucial steps to constructing the best prediction model for the discrimination of *S*-sulfenylation sites from non-*S*-sulfenylation sites. In our study, the predictive model was trained from a variety of features, such as 20D (binary code), AAC, AAPC, BLOSUM62, ASA, PSSM, PWM and physicochemical properties. Five-fold cross-validation was performed for each model and four parameters (Sn, Sp, Acc and MCC) were included as the evaluation criteria. As shown in Table 2, the SVM model trained with PSSM feature yielded the best prediction performance: a sensitivity value of 0.71, specificity of 0.72, accuracy of 0.72, and MCC of 0.30. Slightly below the PSSM model in performance, the sensitivity, specificity, accuracy and MCC of the BLOSUM62 model were 0.68, 0.70, 0.69 and 0.26, respectively. The SVM model constructed with the ASA feature yielded the lowest predictive accuracy (0.61) and relatively lower sensitivity (0.60), specificity (0.66), and MCC (0.14).

**Table 2:** Five-fold cross validation results for SVM models trained with various features individually

Training features	Sn	Sp	Acc	MCC
20D Binary code	0.66	0.68	0.68	0.23
BLOSUM62	0.68	0.70	0.69	0.26
Amino Acid Composition (AAC)	0.64	0.65	0.65	0.19
Amino Acid Pair Composition (AAPC)	0.64	0.67	0.67	0.21
Accessible Surface Area (ASA)	0.60	0.61	0.61	0.14
Secondary structure (SS)	0.56	0.56	0.56	0.08
Position Weight Matrix (PWM)	0.64	0.66	0.66	0.20
Position-specific scoring matrix (PSSM)	0.71	0.72	0.72	0.30

A total of 1145 positive data and 8368 negative data were used in the cross validation process. Sn, sensitivity; Sp, specificity; Acc, accuracy; MCC, Matthews Correlation Coefficient

**Fig. 4:**
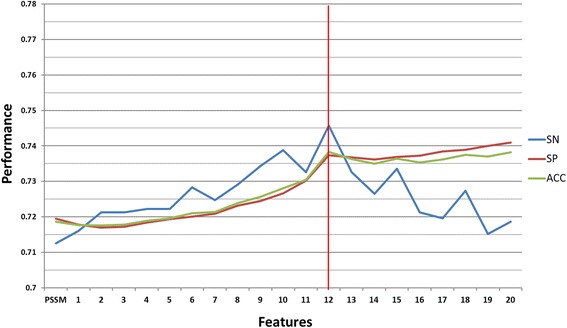
The predictive performance of PSSM model combined with forward selection of the top 20 physicochemical properties

To further investigate the physicochemical properties of S-sulfenylation sites and the adjacent amino acids, a total of 531 physicochemical properties, extracted from version 9.1 of the AAindex, were individually explored. Each physicochemical property with a significant F-score value was assessed by five-fold cross validation in order to evaluate their ability to identify *S*-sulfenylation sites. (Additional file 2: Table S1) shows the top 20 physicochemical properties ranked in a descending order according to their predictive performance. Descriptions of the 531 physicochemical properties from the AAindex indicated that four of the 20 physicochemical properties are associated with hydrophobicity. Overall, the accuracy of each of these features was over 0.6. In order to obtain a better prediction performance, the 20 highest performing physicochemical properties were combined with the best sequence-based feature (PSSM) by forward selection in (Additional file 3: Figure S2). A summary of the resulting performance is illustrated in Fig. 4, while the detailed results are presented in (Additional file 4: Table S2). With the consideration of more physicochemical properties, slight increases in specificity and accuracy were observed, while the sensitivity value fluctuated with different features. The best model yielded a sensitivity of 0.746, specificity of 0.737, accuracy of 0.738 and 0.337 MCC value. This model was built from PSSM and the top 12 most useful physicochemical properties (GUYH850101, JANJ790102, KIDA850101, FASG890101, KARP850101, EISD860102, LEVM760101, GUYH850104, GUYH850102, VINM940103, MIYS990104 and FUKS010111), and appeared to be the most effective at identifying S-sulfenylation sites among all of the models tested so far. Interestingly, three of the 12 selected indices (KIDA850101, FASG890101 and EISD860102) shared a common physicochemical feature, which was hydrophobicity. As analyzed previously, hydrophobicity contributes significantly to the characteristics, structures and functions of these proteins.

### Performance evaluation by independent testing dataset

**Fig. 5:**
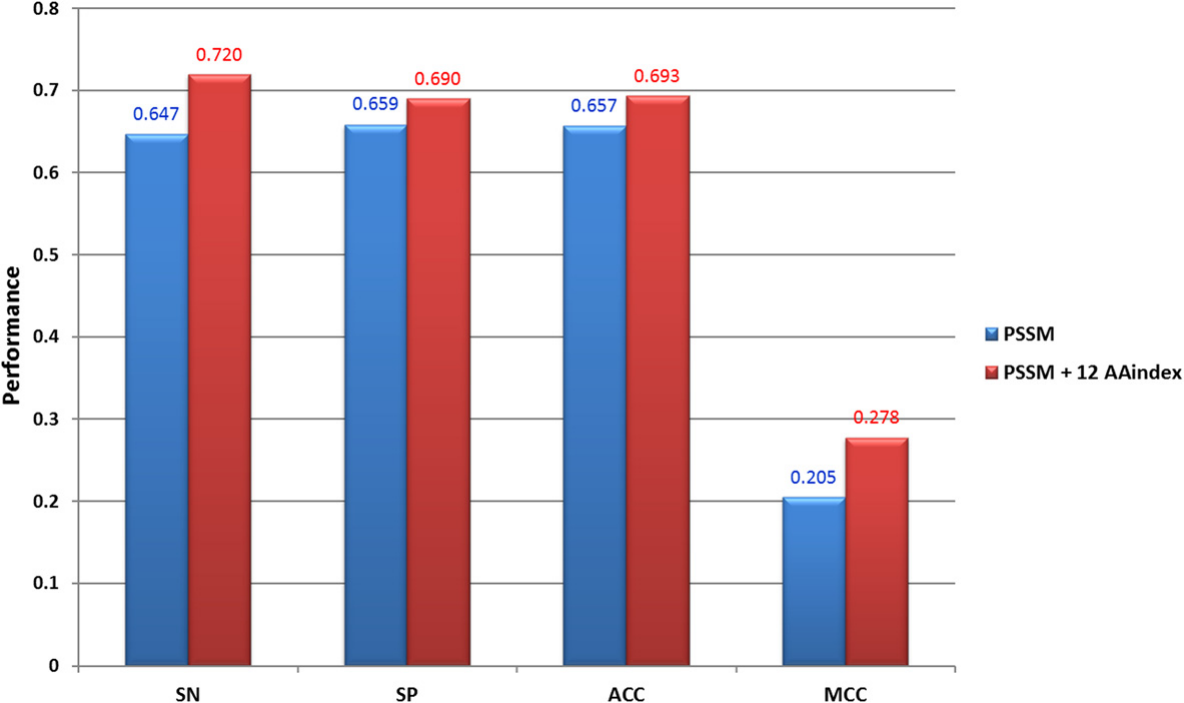
Comparison of the independent testing results between PSSM model and the hybrid model combining PSSM with the top 12 physicochemical properties

For the classification of PTM sites, the prediction performance of the constructed models may be overestimated as a result of the overfitting to a training set. In order to evaluate the real performance of the predictive model, an independent testing data set, which was truly blind to the training data set, was utilized to further test the effectiveness of the selected model (PSSM + 12 AAindex) with the best performance in five-fold cross validation. This independent testing data set consisted of 289 positive data and 2108 negative data extracted from the original data. Figure 5 presents the comparison of the independent testing results between the model trained with the best single feature (PSSM) model and the model trained with the combination of PSSM and the top 12 physicochemical properties. The PSSM model yielded a sensitivity of 0.647, a specificity of 0.659, an accuracy of 0.657, and a MCC value of 0.205. Compared to the PSSM model, the model combining PSSM with the 12 AAindexes generated better sensitivity (0.720), specificity (0.690), accuracy (0.693), and a significantly better MCC (0.278) with *p*-value < 0.05. The detailed independent testing results, including true positive (TP), false negative (FN), true negative (TN) and false positive (FP), are described in (Additional file 5: Table S3). Overall, the model combining PSSM with the 12 AAindexes achieved promising predictive performance on the independent testing data set.

### Functional exploitation of the S-sulfenylated proteins

In this study, the **D**atabase for **A**nnotation, **V**isualization and **I**ntegrated **D**iscovery (DAVID) [40] tool was employed to explore the functions and characteristics of *S*-sulfenylated proteins. (Additional file 6: Table S4) provides the Gene Ontology (GO) [41] annotations associated with the S-sulfenylated proteins examined in this work. According to the GO annotation of biological process, 10.8 % of the proteins were involved in RNA processing (*p*-value = 7.65E-32). In terms of molecular function annotated by GO, most of the proteins seemed to be involved in RNA binding (*p*-value = 1.06E-42) or nucleotide binding (*p*-value = 4.47E-35). With respect to cellular component, these *S*-sulfenylated proteins are mostly distributed in the nuclear lumen (*p*-value = 9.07E-44) and non-membrane-bounded organelles (*p*-value = 1.81E-42). Additionally, pathway analysis showed that *S*-sulfenylated proteins are involved in a variety of pathways in (Additional file 7: Table S5). Of note, 27 of the 1096 *S*-sulfenylated proteins appeared to be strongly associated with the mechanisms underlying *Escherichia coli* infection.

It has been reported that a protein-interacting domain usually recognizes a short peptide motif on the target protein but does not bind stably until the peptide has undergone appropriate PTM [42–44]; this can create binding sites for specific protein-interaction domains that work together to carry out a specific cellular function [45]. The redox state and chemical modification of the thiol group of cysteine residues facilitate its interaction with various proteins to regulate a variety of intracellular and intercellular events [46]. Thus, information regarding the functional domains could be utilized to infer the functional roles of *S*-sulfenylation sites located in a specific protein domain. InterPro [47] is an integrated resource that provides "signatures" such as protein families, domains, and functional sites. Our investigation of the protein domains revealed that the thioredoxin-like fold domain is the most abundant functional domain in *S*-sulfenylated proteins in (Additional file 8: Table S6).

### Distinguishing *S*-sulfenylation from *S*-nitrosylation or *S*-glutathionylation

**Fig. 6:**
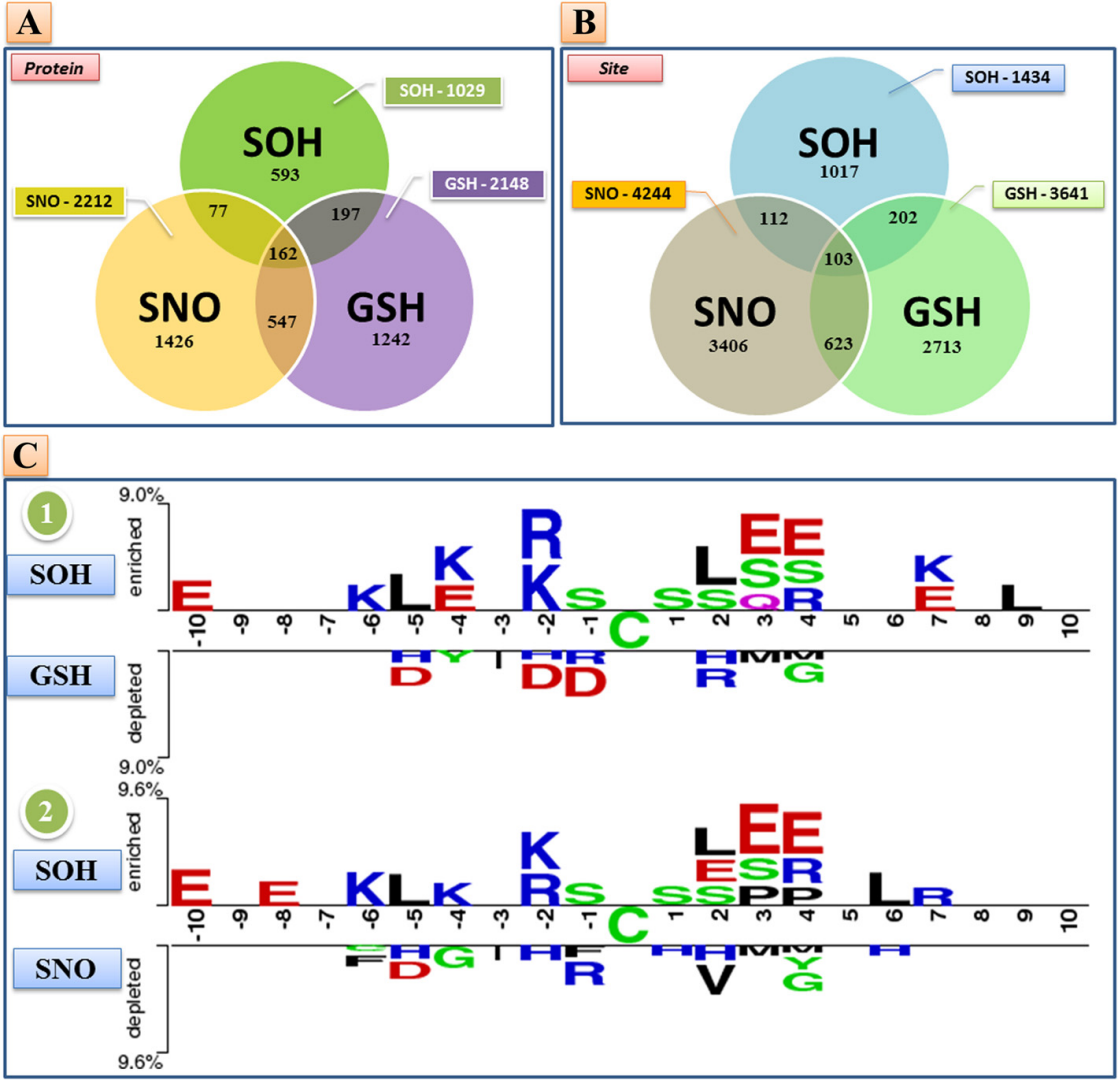
Discrimination of *S*-sulfenylation sites from *S*-nitrosylation and *S*-glutathionylation sites. **a** Number of duplicate proteins among S-sulfenylation, S-nitrosylation and S-glutathionylation; (**b**) Number of duplicate sites among S-sulfenylation, S-nitrosylation and S-glutathionylation; (**c**) Significant differences in position-specific compositions among three PTMs as identified by TwoSampleLogo

The primary purpose of this study was to effectively identify *S*-sulfenylation sites from a large-scale proteome data with modified cysteine residues. However, an increasing number of cysteine-based redox modifications, such as *S-*sulfenylation, *S*-nitrosylation and *S*-glutathionylation, were reported to share the same cysteine targets [48]. Therefore, investigating the sequence characteristics among these cysteine-based PTMs is necessary for achieving reliable detection of *S*-sulfenylation sites. According to experimentally verified data obtained from dbSNO [49, 50], there exist 2212 *S*-nitrosylated proteins containing 4244 nitrosylation sites. In addition, a total of 2148 S-glutathionylated proteins harboring 3641 S-glutathionylation sites were obtained from dbGSH [51]. To avoid redundancy in data as a result of homologous sequences, sequence fragments were deleted if they were identified with 100 % similarity across the three datasets. Overall, 162 (15.74 %) of the 1029 *S*-sulfenylated proteins may undergo all three types of PTMs (Fig. 6A). In addition, percentages of the 1029 *S*-sulfenylated proteins that were also modified by *S*-nitrosylation and *S*-glutathionylation are approximately 23.23 % and 34.89 %, respectively. Investigation of the 1434 *S*-sulfenylated cysteine residues revealed that only 103 sites (7.18 %) could undergo all the three PTMs. As illustrated in Fig. 6B, the percentage of sites that are susceptible to both *S*-sulfenylation and *S*-glutathionylation (21.27 %) appeared to be higher than those that can be modified by *S*-sulfenylation and *S*-nitrosylation (14.99 %).

Taking advantage of the TwoSampleLogo, we uncovered the potential consensus motifs that may distinguish *S*-sulfenylation sites from *S*-nitrosylation or *S*-glutathionylation sites. As represented in Fig. 6C, the figure on the top panel describes notable differences in the position-specific compositions between *S*-sulfenylation and *S*-glutathionylation, while the figure on the bottom panel shows the potential amino acid composition difference between *S*-sulfenylation and *S*-nitrosylation sites. It seemed that *S*-sulfenylation sites could be recognized based on specific positions such as −10, −6, −5, −2, and +2 to +4 (*p*-value < 0.01). This investigation indicated a consistent composition of amino acids with Fig. 2D, particularly at positions −6, −2, +3 and +4. The frequency of positively charged groups, including R (Arginine) and K (Lysine), appeared to be significant at position −6 and −2, and that the occurrence of E (Glutamic acid) belonging to negatively charged residue was also apparent at positions +3 and +4.

## Conclusions

This study describes a systematic investigation on the experimentally verified *S*-sulfenylation sites based on amino acid composition. The analysis of position-specific amino acids composition revealed that the most pronounced feature of *S*-sulfenylation sites is the abundance of positively charged amino acids (K and R) at surrounding positions: −10, −8 ~ −6, −4, −2, and +4 to +8. However, the depletion of positively charged residues was observed at positions −1, +1 and +2 that are close to the *S*-sulfenylation sites. Additionally, a noticeable abundance of negatively charged resides (E) was found at positions −3, +1, +3, +4. This investigation suggested that the amino acid position relative to one another in the sequence may play a vital role in discriminating between *S*-sulfenylation and non-*S*-sulfenylation sites. The comparison of position-specific amino acid composition between *S*-sulfenylation and non-*S*-sulfenylation sites also implicated that, for *S*-sulfenylation sites, distant amino acids around position −7 and +7, which may be close to *S*-sulfenylated cysteines in three-dimensional structure, harbor a notable abundance of positively charged amino acids (K and R).

In this work, the solvent accessibility and physicochemical properties were considered in the characterization of *S*-sulfenylation sites. The *S*-sulfenylation sites have a higher solvent accessibility, especially at the positions −7, +1, +2 and +4. According to the F-score measurements on 531 physicochemical properties, 20 physicochemical properties were revealed to have statistically significant differences between *S*-sulfenylation and non-*S*-sulfenylation sites. Based on the evaluation by five-fold cross-validation, the model trained with the combined features of evolutionary information (PSSM) and 12 physicochemical properties yielded the best prediction performance. Overall, our study not only determined the best prediction model for the identification of S-sulfenylation sites, but also discovered several characteristics and functions regarding S-sulfenylated proteins. Hydrophobic characteristics of S-sulfenylated proteins seem to be crucial for the understanding of their functions. Moreover, amino acids R (Arginine), E (Glutaminc acid), and K (Lysine) may be the potential consensus motifs for specific S-sulfenylated sites.

## Additional files

Additional file 1: Figure S1. Analytical flowchart of removing homologous sequences in training dataset and independent testing dataset. (DOCX 242 kb) Additional file 2: Table S1. Top 20 best performing physicochemical properties as evaluated by five-fold cross-validation. (DOCX 17 kb) Additional file 3: Figure S2. Methods for combining PSSM with physicochemical properties by forward selection. (DOCX 126 kb) Additional file 4: Table S2. Five-fold cross-validation performance of combining PSSM with the top 20 physicochemical properties by forward selection. (DOCX 17 kb) Additional file 5: Table S3. Comparison of independent testing results between the PSSM model and the hybrid model combining PSSM with the top 12 physicochemical properties. (DOCX 14 kb) Additional file 6: Table S4. Top 10 most enriched GO categories associated with *S*-sulfenylated proteins (*p* < 0.01). (DOCX 16 kb) Additional file 7: Table S5. Distribution of KEGG pathway annotations for *S*-sulfenylated proteins. (DOCX 15 kb) Additional file 8: Table S6. Distribution of InterPro functional domains covering *S*-sulfenylated sites. (DOCX 17 kb)

## References

[1] Leonard SE, Carroll KS (2011). Chemical 'omics' approaches for understanding protein cysteine oxidation in biology. Curr Opin Chem Biol.

[2] Poole LB, Nelson KJ (2008). Discovering mechanisms of signaling-mediated cysteine oxidation. Curr Opin Chem Biol.

[3] Wani R, Qian J, Yin L, Bechtold E, King SB, Poole LB, Paek E, Tsang AW, Furdui CM (2011). Isoform-specific regulation of Akt by PDGF-induced reactive oxygen species. Proc Natl Acad Sci U S A.

[4] Roos G, Messens J (2011). Protein sulfenic acid formation: From cellular damage to redox regulation. Free Radic Biol Med.

[5] Leonard SE, Reddie KG, Carroll KS (2009). Mining the thiol proteome for sulfenic acid modifications reveals New targets for oxidation in cells. ACS Chem Biol.

[6] Weerapana E, Wang C, Simon GM, Richter F, Khare S, Dillon MBD, Bachovchin DA, Mowen K, Baker D, Cravatt BF (2010). Quantitative reactivity profiling predicts functional cysteines in proteomes. Nature.

[7] Wang C, Weerapana E, Blewett MM, Cravatt BF (2014). A chemoproteomic platform to quantitatively map targets of lipid-derived electrophiles. Nat Methods.

[8] Szychowski J, Mahdavi A, Hodas JJL, Bagert JD, Ngo JT, Landgraf P, Dieterich DC, Schuman EM, Tirrell DA (2010). Cleavable biotin probes for labeling of biomolecules via azide-alkyne cycloaddition. J Am Chem Soc.

[9] Qian Y, Martell J, Pace NJ, Ballard TE, Johnson DS, Weerapana E (2013). An isotopically tagged azobenzene-based cleavable linker for quantitative proteomics. Chembiochem.

[10] Zheng T, Jiang H, Wu P (2013). Single-stranded DNA as a cleavable linker for bioorthogonal click chemistry-based proteomics. Bioconjug Chem.

[11] Yang J, Gupta V, Carroll KS, Liebler DC (2014). Site-specific mapping and quantification of protein S-sulphenylation in cells. Nat Commun.

[12] M-a S, Wang Y, Cheng H, Zhang Q, Ge W, Guo D (2012). RedoxDB-a curated database for experimentally verified protein oxidative modification. Bioinformatics.

[13] Mucchielli-Giorgi MHM, Hazout S, Tuffery P (2002). Predicting the disulfide bonding state of cysteines using protein descriptors. Proteins-Structure Function and Genetics.

[14] Chang C-C, Lin C-J. LIBSVM: A Library for Support Vector Machines. ACM Trans Intell Syst Technol. 2011;2(3):27:1–27:27.

[15] UniProt C (2015). UniProt: a hub for protein information. Nucleic Acids Res.

[16] Furdui CM, Poole LB (2014). Chemical approaches to detect and analyze protein sulfenic acids. Mass Spectrom Rev.

[17] Shien D-M, Lee T-Y, Chang W-C, Hsu JB-K, Horng J-T, Hsu P-C, Wang T-Y, Huang H-D (2009). Incorporating structural characteristics for identification of protein methylation sites. J Comput Chem.

[18] Tatusova TA, Madden TL (1999). BLAST 2 Sequences, a new tool for comparing protein and nucleotide sequences. FEMS Microbiol Lett.

[19] Wong YH, Lee TY, Liang HK, Huang CM, Wang TY, Yang YH, Chu CH, Huang HD, Ko MT, Hwang JK (2007). KinasePhos 2.0: a web server for identifying protein kinase-specific phosphorylation sites based on sequences and coupling patterns. Nucleic Acids Res.

[20] Henikoff S, Henikoff JG (1992). Amino acid substitution matrices from protein blocks. Proc Natl Acad Sci U S A.

[21] Chang W-C, Lee T-Y, Shien D-M, Hsu JB-K, Horng J-T, Hsu P-C, Wang T-Y, Huang H-D, Pan R-L (2009). Incorporating support vector machine for identifying protein tyrosine sulfation sites. J Comput Chem.

[22] Altschul SF, Madden TL, Schaffer AA, Zhang J, Zhang Z, Miller W, Lipman DJ (1997). Gapped BLAST and PSI-BLAST: a new generation of protein database search programs. Nucleic Acids Res.

[23] Pang CNI, Hayen A, Wilkins MR (2007). Surface accessibility of protein post-translational modifications. J Proteome Res.

[24] Ahmad S, Gromiha MM, Sarai A (2003). RVP-net: online prediction of real valued accessible surface area of proteins from single sequences. Bioinformatics.

[25] Ahmad S, Gromiha MM, Sarai A (2003). Real value prediction of solvent accessibility from amino acid sequence. Proteins-Structure Function and Genetics.

[26] Rose PW, Prlic A, Bi C, Bluhm WF, Christie CH, Dutta S, Green RK, Goodsell DS, Westbrook JD, Woo J (2015). The RCSB Protein Data Bank: views of structural biology for basic and applied research and education. Nucleic Acids Res.

[27] McGuffin LJ, Bryson K, Jones DT (2000). The PSIPRED protein structure prediction server. Bioinformatics.

[28] Kawashima S, Pokarowski P, Pokarowska M, Kolinski A, Katayama T, Kanehisa M (2008). AAindex: amino acid index database, progress report 2008. Nucleic Acids Res.

[29] Nguyen VN, Huang KY, Huang CH, Chang TH, Bretana N, Lai K, Weng J, Lee TY (2015). Characterization and identification of ubiquitin conjugation sites with E3 ligase recognition specificities. BMC bioinformatics.

[30] Su MG, Huang KY, Lu CT, Kao HJ, Chang YH, Lee TY (2014). topPTM: a new module of dbPTM for identifying functional post-translational modifications in transmembrane proteins. Nucleic Acids Res.

[31] Lee TY, Chen YJ, Lu TC, Huang HD, Chen YJ. SNOSite: Exploiting Maximal Dependence Decomposition to Identify Cysteine S-Nitrosylation with Substrate Site Specificity. Plos One. 2011;6(7):e21849. 10.1371/journal.pone.0021849 PMC3137596 21789187

[32] Lee TY, Chen SA, Hung HY, Ou YY. Incorporating distant sequence features and radial basis function networks to identify ubiquitin conjugation sites. Plos One. 2011;6(3):e17331. 10.1371/journal.pone.0017331 PMC3052307 21408064

[33] Hsu JBK, Bretana NA, Lee TY, Huang HD. Incorporating Evolutionary Information and Functional Domains for Identifying RNA Splicing Factors in Humans. Plos One. 2011;6(11):e27567. 10.1371/journal.pone.0027567 PMC3217973 22110674

[34] Lin C-J, Chen Y-W. Combining SVMs with various feature selection strategies. Feature Extraction. 2003;207:315–24.

[35] Huang HD, Lee TY, Tzeng SW, Horng JT. KinasePhos: a web tool for identifying protein kinase-specific phosphorylation sites. Nucleic Acids Res. 2005;33(Web Server issue):W226-229. 10.1093/nar/gki471 PMC1160232 15980458

[36] Lu C-T, Chen S-A, Bretana NA, Cheng T-H, Lee T-Y (2011). Carboxylator: incorporating solvent-accessible surface area for identifying protein carboxylation sites. J Comput Aided Mol Des.

[37] Bui VM, Lu CT, Ho TT, Lee TY. MDD-SOH: Exploiting maximal dependence decomposition to identify S-sulfenylation sites with substrate motifs. Bioinformatics. 2015. 10.1093/bioinformatics/btv558 26411868

[38] Crooks GE, Hon G, Chandonia JM, Brenner SE (2004). WebLogo: A sequence logo generator. Genome Res.

[39] Vacic V, Iakoucheva LM, Radivojac P (2006). Two Sample Logo: a graphical representation of the differences between two sets of sequence alignments. Bioinformatics.

[40] Dennis G, Sherman BT, Hosack DA, Yang J, Gao W, Lane HC, Lempicki RA (2003). DAVID: Database for annotation, visualization, and integrated discovery. Genome Biol.

[41] Consortium TGO (2011). The Gene Ontology: enhancements for 2011. Nucleic Acids Res.

[42] Huang KY, Wu HY, Chen YJ, Lu CT, Su MG, Hsieh YC, et al. RegPhos 2.0: an updated resource to explore protein kinase-substrate phosphorylation networks in mammals. Database : the journal of biological databases and curation. 2014;2014(0):bau034. 10.1093/database/bau034 PMC3999940 24771658

[43] Lu CT, Huang KY, Su MG, Lee TY, Bretana NA, Chang WC, Chen YJ, Chen YJ, Huang HD (2013). dbPTM 3.0: an informative resource for investigating substrate site specificity and functional association of protein post-translational modifications. Nucleic Acids Res.

[44] Lee TY, Bo-Kai Hsu J, Chang WC, Huang HD (2011). RegPhos: a system to explore the protein kinase-substrate phosphorylation network in humans. Nucleic Acids Res.

[45] Seet BT, Dikic I, Zhou MM, Pawson T (2006). Reading protein modifications with interaction domains. Nat Rev Mol Cell Biol.

[46] Aranda E, Lopez-Pedrera C, De La Haba-Rodriguez JR, Rodriguez-Ariza A (2012). Nitric oxide and cancer: the emerging role of S-nitrosylation. Curr Mol Med.

[47] Hunter S, Jones P, Mitchell A, Apweiler R, Attwood TK, Bateman A, Bernard T, Binns D, Bork P, Burge S (2011). InterPro in 2011: new developments in the family and domain prediction database. Nucleic Acids Res.

[48] Couturier J, Chibani K, Jacquot JP, Rouhier N (2013). Cysteine-based redox regulation and signaling in plants. Frontiers in plant science.

[49] Chen Y-J, Lu C-T, Su M-G, Huang K-Y, Ching W-C, Yang H-H, Liao Y-C, Chen Y-J, Lee T-Y (2015). dbSNO 2.0: a resource for exploring structural environment, functional and disease association and regulatory network of protein S-nitrosylation. Nucleic Acids Res.

[50] Lee TY, Chen YJ, Lu CT, Ching WC, Teng YC, Huang HD, Chen YJ (2012). dbSNO: a database of cysteine S-nitrosylation. Bioinformatics.

[51] Chen YJ, Lu CT, Lee TY, Chen YJ (2014). dbGSH: a database of S-glutathionylation. Bioinformatics.

