# Supplementary material for: SOHSite: incorporating evolutionary information and physicochemical properties to identify protein S-sulfenylation sites
Source: BMC Genomics. 2016 Jan 11;17(Suppl 1):9. doi: 10.1186/s12864-015-2299-1 (PMC4895302; doi:10.1186/s12864-015-2299-1)
Supplement: Additional file 1: Figure S1. — Analytical flowchart of removing homologous sequences in training dataset and independent testing dataset. (DOCX 242 kb) [file 12864_2015_2299_MOESM1_ESM.docx]

**
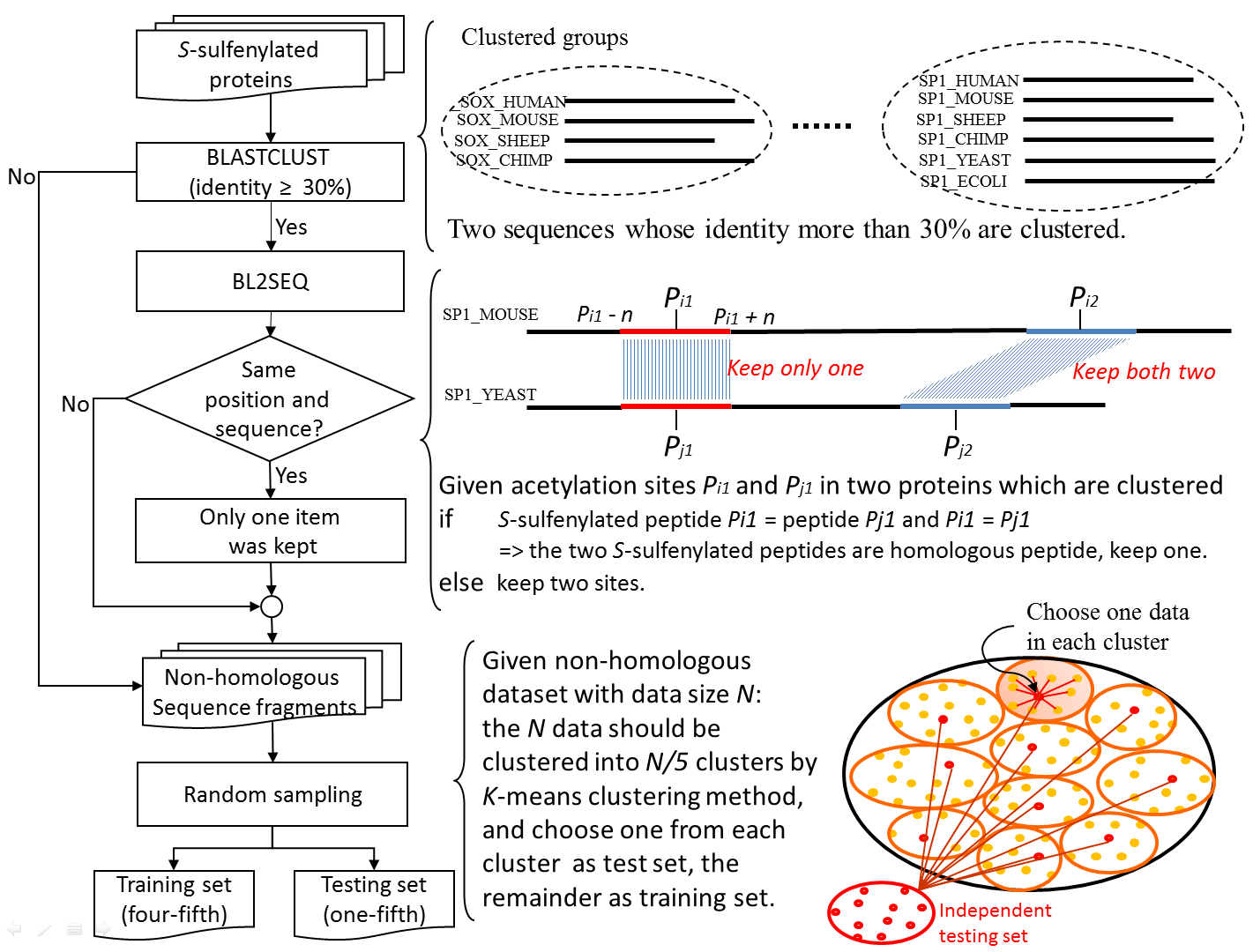
**

**Figure S1. Analytical flowchart of removing homologous sequences in training dataset and independent testing dataset.**
