# Supplementary material for: SOHSite: incorporating evolutionary information and physicochemical properties to identify protein S-sulfenylation sites
Source: BMC Genomics. 2016 Jan 11;17(Suppl 1):9. doi: 10.1186/s12864-015-2299-1 (PMC4895302; doi:10.1186/s12864-015-2299-1)
Supplement: Additional file 2: Table S1. — Top 20 best performing physicochemical properties as evaluated by five-fold cross-validation. (DOCX 17 kb) [file 12864_2015_2299_MOESM2_ESM.docx]

**Table S1. Top 20 best performing physicochemical properties as evaluated by five-fold cross-validation.**

| **AAindex ID** | **Description** | **Sn** | **Sp** | **Acc** | **MCC** |
| --- | --- | --- | --- | --- | --- |
| GUYH850101 | Partition energy | 0.624 | 0.629 | 0.629 | 0.168 |
| JANJ790102 | Transfer free energy | 0.623 | 0.629 | 0.628 | 0.167 |
| KIDA850101 | Hydrophobicity-related index | 0.620 | 0.626 | 0.625 | 0.163 |
| FASG890101 | Hydrophobicity index | 0.619 | 0.626 | 0.625 | 0.163 |
| KARP850101 | Flexibility parameter for no rigid neighbors | 0.622 | 0.624 | 0.624 | 0.163 |
| EISD860102 | Atom-based hydrophobic moment | 0.619 | 0.625 | 0.624 | 0.162 |
| LEVM760101 | Hydrophobic parameter | 0.618 | 0.623 | 0.623 | 0.160 |
| GUYH850104 | Number of hydrogen bond donors | 0.616 | 0.623 | 0.622 | 0.158 |
| GUYH850102 | Apparent partition energies calculated from Wertz-Scheraga index | 0.619 | 0.622 | 0.622 | 0.160 |
| VINM940103 | Normalized flexibility parameters (B-values) for each residue surrounded by one rigid neighbours | 0.619 | 0.622 | 0.621 | 0.160 |
| MIYS990104 | Optimized relative partition energies - method C | 0.612 | 0.622 | 0.621 | 0.156 |
| FUKS010111 | Entire chain composition of amino acids in extracellular proteins of mesophiles (percent) | 0.610 | 0.622 | 0.621 | 0.154 |
| KRIW790102 | Fraction of site occupied by water | 0.615 | 0.621 | 0.620 | 0.156 |
| FINA910104 | Helix termination parameter at posision j+1 | 0.615 | 0.620 | 0.619 | 0.155 |
| CEDJ970102 | Composition of amino acids in anchored proteins (percent) | 0.610 | 0.620 | 0.619 | 0.152 |
| KLEP840101 | Net charge | 0.610 | 0.619 | 0.617 | 0.151 |
| ROSG850102 | Mean fractional area loss | 0.610 | 0.618 | 0.617 | 0.151 |
| KRIW790101 | Side chain interaction parameter | 0.612 | 0.617 | 0.617 | 0.152 |
| JANJ780101 | Average accessible surface area | 0.616 | 0.616 | 0.616 | 0.153 |
| HOPT810101 | Hydrophilicity value | 0.608 | 0.617 | 0.616 | 0.149 |
