# Supplementary figures and images for: SOHSite: incorporating evolutionary information and physicochemical properties to identify protein S-sulfenylation sites
Source: BMC Genomics. 2016 Jan 11;17(Suppl 1):9. doi: 10.1186/s12864-015-2299-1 (PMC4895302; doi:10.1186/s12864-015-2299-1)

**
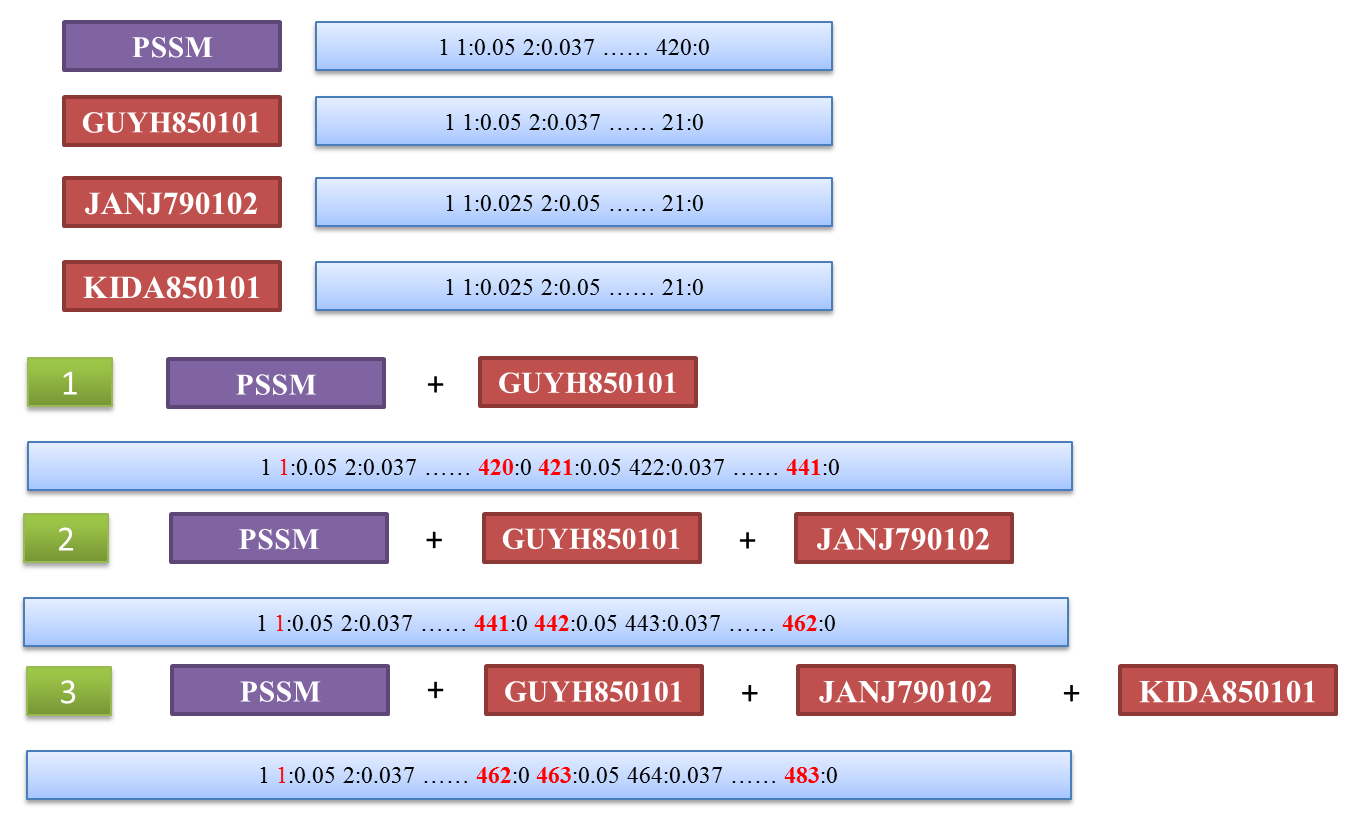
**

**Figure S2. Methods for combining PSSM with physicochemical properties by forward selection.**

Supplement: Additional file 3: Figure S2. — Methods for combining PSSM with physicochemical properties by forward selection. (DOCX 126 kb) [file 12864_2015_2299_MOESM3_ESM.docx]
