# Supplementary material for: SOHSite: incorporating evolutionary information and physicochemical properties to identify protein S-sulfenylation sites
Source: BMC Genomics. 2016 Jan 11;17(Suppl 1):9. doi: 10.1186/s12864-015-2299-1 (PMC4895302; doi:10.1186/s12864-015-2299-1)
Supplement: Additional file 4: Table S2. — Five-fold cross-validation performance of combining PSSM with the top 20 physicochemical properties by forward selection. (DOCX 17 kb) [file 12864_2015_2299_MOESM4_ESM.docx]

**Table S2. Five-fold cross-validation performance of combining PSSM with the top 20 physicochemical properties by forward selection.**

| **Feature** | **TP** | **FP** | **TN** | **FN** | **Sn** | **Sp** | **Acc** | **MCC** |
| --- | --- | --- | --- | --- | --- | --- | --- | --- |
| PSSM | 816 | 2347 | 6021 | 329 | 0.713 | 0.720 | 0.719 | 0.299 |
| 1 | 820 | 2360 | 6008 | 325 | 0.716 | 0.718 | 0.718 | 0.299 |
| 2 | 826 | 2367 | 6001 | 319 | 0.721 | 0.717 | 0.718 | 0.302 |
| 3 | 826 | 2365 | 6003 | 319 | 0.721 | 0.717 | 0.718 | 0.302 |
| 4 | 827 | 2355 | 6013 | 318 | 0.722 | 0.719 | 0.719 | 0.304 |
| 5 | 827 | 2348 | 6020 | 318 | 0.722 | 0.719 | 0.720 | 0.305 |
| 6 | 834 | 2342 | 6026 | 311 | 0.728 | 0.720 | 0.721 | 0.309 |
| 7 | 830 | 2335 | 6033 | 315 | 0.725 | 0.721 | 0.721 | 0.308 |
| 8 | 835 | 2316 | 6052 | 310 | 0.729 | 0.723 | 0.724 | 0.313 |
| 9 | 841 | 2305 | 6063 | 304 | 0.734 | 0.725 | 0.726 | 0.317 |
| 10 | 846 | 2287 | 6081 | 299 | 0.739 | 0.727 | 0.728 | 0.322 |
| 11 | 839 | 2257 | 6111 | 306 | 0.733 | 0.730 | 0.731 | 0.322 |
| 12 | 854 | 2197 | 6171 | 291 | **0.746** | **0.737** | **0.738** | **0.337** |
| 13 | 839 | 2202 | 6166 | 306 | 0.733 | 0.737 | 0.736 | 0.328 |
| 14 | 832 | 2207 | 6161 | 313 | 0.727 | 0.736 | 0.735 | 0.323 |
| 15 | 840 | 2201 | 6167 | 305 | 0.734 | 0.737 | 0.737 | 0.328 |
| 16 | 826 | 2198 | 6170 | 319 | 0.721 | 0.737 | 0.735 | 0.321 |
| 17 | 824 | 2188 | 6180 | 321 | 0.720 | 0.739 | 0.736 | 0.321 |
| 18 | 833 | 2184 | 6184 | 312 | 0.728 | 0.739 | 0.738 | 0.326 |
| 19 | 819 | 2175 | 6193 | 326 | 0.715 | 0.741 | 0.737 | 0.319 |
| 20 | 823 | 2167 | 6201 | 322 | 0.719 | 0.741 | 0.738 | 0.322 |
