# Supplementary material for: SOHSite: incorporating evolutionary information and physicochemical properties to identify protein S-sulfenylation sites
Source: BMC Genomics. 2016 Jan 11;17(Suppl 1):9. doi: 10.1186/s12864-015-2299-1 (PMC4895302; doi:10.1186/s12864-015-2299-1)
Supplement: Additional file 5: Table S3. — Comparison of independent testing results between the PSSM model and the hybrid model combining PSSM with the top 12 physicochemical properties. (DOCX 14 kb) [file 12864_2015_2299_MOESM5_ESM.docx]

**Table S3. Comparison of independent testing results between the PSSM model and the hybrid model combining PSSM with the top 12 physicochemical properties.**

| **Model** | **TP** | **FP** | **TN** | **FN** | **Sn** | **Sp** | **Acc** | **MCC** |
| --- | --- | --- | --- | --- | --- | --- | --- | --- |
| PSSM | 187 | 719 | 1389 | 102 | 0.647 | 0.659 | 0.657 | 0.205 |
| PSSM + 12 AAindex* | 208 | 654 | 1454 | 81 | 0.720 | 0.690 | 0.693 | 0.278 |

*Combining PSSM with 12 physicochemical properties (GUYH850101, JANJ790102, KIDA850101, FASG890101, KARP850101, EISD860102, LEVM760101, GUYH850104, GUYH850102, VINM940103, MIYS990104 and FUKS010111)
