# Supplementary material for: SOHSite: incorporating evolutionary information and physicochemical properties to identify protein S-sulfenylation sites
Source: BMC Genomics. 2016 Jan 11;17(Suppl 1):9. doi: 10.1186/s12864-015-2299-1 (PMC4895302; doi:10.1186/s12864-015-2299-1)
Supplement: Additional file 6: Table S4. — Top 10 most enriched GO categories associated with S-sulfenylated proteins (p < 0.01). (DOCX 16 kb) [file 12864_2015_2299_MOESM6_ESM.docx]

**Table S4. Top 10 distributions of GO annotations for *S*-sulfenylated proteins by DAVID analysis (*p*-value < 0.01).**

| **GO ID** | **GO Terms** | **Number of proteins** | **Total**  **(%)** | ***p*-value** |
| --- | --- | --- | --- | --- |
| **GO Biological Processes** | | | | |
| GO:0006396 | RNA processing | 113 | 10.80 | 7.65E-32 |
| GO:0022613 | ribonucleoprotein complex biogenesis | 49 | 4.68 | 4.88E-19 |
| GO:0046907 | intracellular transport | 100 | 9.56 | 5.83E-18 |
| GO:0042254 | ribosome biogenesis | 39 | 3.73 | 7.93E-18 |
| GO:0034660 | ncRNA metabolic process | 53 | 5.07 | 3.74E-17 |
| GO:0006412 | translation | 63 | 6.02 | 7.47E-16 |
| GO:0034470 | ncRNA processing | 45 | 4.30 | 2.75E-15 |
| GO:0006414 | translational elongation | 32 | 3.06 | 1.55E-14 |
| GO:0016072 | rRNA metabolic process | 31 | 2.96 | 2.41E-14 |
| GO:0016071 | mRNA metabolic process | 64 | 6.12 | 4.32E-14 |
| **GO Molecular Function** | | | | |
| GO:0003723 | RNA binding | 148 | 14.15 | 1.06E-42 |
| GO:0000166 | nucleotide binding | 273 | 26.10 | 4.47E-35 |
| GO:0032553 | ribonucleotide binding | 217 | 20.75 | 4.54E-25 |
| GO:0032555 | purine ribonucleotide binding | 217 | 20.75 | 4.54E-25 |
| GO:0017076 | purine nucleotide binding | 223 | 21.32 | 7.09E-25 |
| GO:0003924 | GTPase activity | 49 | 4.68 | 4.36E-16 |
| GO:0005524 | ATP binding | 162 | 15.49 | 6.01E-15 |
| GO:0001882 | nucleoside binding | 172 | 16.44 | 8.53E-15 |
| GO:0032559 | adenyl ribonucleotide binding | 163 | 15.58 | 9.19E-15 |
| GO:0001883 | purine nucleoside binding | 171 | 16.35 | 9.65E-15 |
| **GO Cellular Component** | | | | |
| GO:0031981 | nuclear lumen | 220 | 21.03 | 9.07E-44 |
| GO:0043228 | non-membrane-bounded organelle | 311 | 29.73 | 1.81E-42 |
| GO:0043232 | intracellular non-membrane-bounded organelle | 311 | 29.73 | 1.81E-42 |
| GO:0031974 | membrane-enclosed lumen | 252 | 24.09 | 3.67E-42 |
| GO:0070013 | intracellular organelle lumen | 244 | 23.33 | 2.40E-41 |
| GO:0043233 | organelle lumen | 246 | 23.52 | 1.29E-40 |
| GO:0005829 | cytosol | 203 | 19.40 | 2.48E-40 |
| GO:0030529 | ribonucleoprotein complex | 114 | 10.90 | 1.71E-36 |
| GO:0005730 | nucleolus | 125 | 11.95 | 1.64E-30 |
| GO:0005654 | nucleoplasm | 124 | 11.85 | 1.33E-20 |
