# Supplementary material for: SOHSite: incorporating evolutionary information and physicochemical properties to identify protein S-sulfenylation sites
Source: BMC Genomics. 2016 Jan 11;17(Suppl 1):9. doi: 10.1186/s12864-015-2299-1 (PMC4895302; doi:10.1186/s12864-015-2299-1)
Supplement: Additional file 7: Table S5. — Distribution of KEGG pathway annotations for S-sulfenylated proteins. (DOCX 15 kb) [file 12864_2015_2299_MOESM7_ESM.docx]

**Table S5. Distribution of KEGG pathway annotations for *S*-sulfenylated proteins.**

| **#** | **KEGG pathway map ID** | **Pathway terms** | **Number of proteins** | **Total**  **(%)** | ***p*-value** |
| --- | --- | --- | --- | --- | --- |
| 1 | hsa05130 | Pathogenic Escherichia coli infection | 27 | 2.58 | 1.54E-15 |
| 2 | hsa03010 | Ribosome | 26 | 2.49 | 9.85E-10 |
| 3 | hsa04540 | Gap junction | 22 | 2.10 | 8.94E-07 |
| 4 | hsa00010 | Glycolysis / Gluconeogenesis | 15 | 1.43 | 6.99E-05 |
| 5 | hsa04530 | Tight junction | 24 | 2.29 | 7.64E-05 |
| 6 | hsa03040 | Spliceosome | 23 | 2.20 | 8.40E-05 |
| 7 | hsa03018 | RNA degradation | 13 | 1.24 | 6.33E-04 |
| 8 | hsa04520 | Adherens junction | 15 | 1.43 | 1.09E-03 |
| 9 | hsa03050 | Proteasome | 11 | 1.05 | 1.63E-03 |
| 10 | hsa00620 | Pyruvate metabolism | 10 | 0.96 | 1.84E-03 |
| 11 | hsa00270 | Cysteine and methionine metabolism | 9 | 0.86 | 2.42E-03 |
| 12 | hsa04510 | Focal adhesion | 27 | 2.58 | 2.53E-03 |
| 13 | hsa00030 | Pentose phosphate pathway | 7 | 0.67 | 7.60E-03 |
| 14 | hsa05416 | Viral myocarditis | 12 | 1.15 | 1.25E-02 |
| 15 | hsa04110 | Cell cycle | 17 | 1.63 | 1.78E-02 |
| 16 | hsa00640 | Propanoate metabolism | 7 | 0.67 | 2.53E-02 |
| 17 | hsa00970 | Aminoacyl-tRNA biosynthesis | 8 | 0.76 | 2.63E-02 |
| 18 | hsa00051 | Fructose and mannose metabolism | 7 | 0.67 | 3.33E-02 |
| 19 | hsa04722 | Neurotrophin signaling pathway | 16 | 1.53 | 3.37E-02 |
| 20 | hsa04810 | Regulation of actin cytoskeleton | 24 | 2.29 | 3.72E-02 |
