# Supplementary material for: SOHSite: incorporating evolutionary information and physicochemical properties to identify protein S-sulfenylation sites
Source: BMC Genomics. 2016 Jan 11;17(Suppl 1):9. doi: 10.1186/s12864-015-2299-1 (PMC4895302; doi:10.1186/s12864-015-2299-1)
Supplement: Additional file 8: Table S6. — Distribution of InterPro functional domains covering S-sulfenylated sites. (DOCX 17 kb) [file 12864_2015_2299_MOESM8_ESM.docx]

**Table S6. Distribution of InterPro functional domains covering *S*-sulfenylated sites.**

| **#** | **InterPro ID** | **InterPro domain terms** | **Number of sites** | **Total**  **(%)** | ***p*-value** |
| --- | --- | --- | --- | --- | --- |
| 1 | IPR012336 | Thioredoxin-like fold | 25 | 1.74 | 7.47E-06 |
| 2 | IPR012335 | Thioredoxin fold | 24 | 1.67 | 9.78E-06 |
| 3 | IPR012677 | Nucleotide-binding, alpha-beta plait | 24 | 1.67 | 1.86E-09 |
| 4 | IPR005225 | Small GTP-binding protein | 23 | 1.6 | 1.67E-05 |
| 5 | IPR000504 | RNA recognition motif, RNP-1 | 20 | 1.39 | 3.81E-10 |
| 6 | IPR001806 | Ras GTPase | 17 | 1.19 | 3.27E-03 |
| 7 | IPR000217 | Tubulin | 16 | 1.12 | 1.04E-13 |
| 8 | IPR008280 | Tubulin/FtsZ, C-terminal | 16 | 1.12 | 1.04E-13 |
| 9 | IPR013753 | Ras | 16 | 1.12 | 1.48E-03 |
| 10 | IPR011009 | Protein kinase-like | 15 | 1.05 | 1.67E-05 |
| 11 | IPR011046 | WD40 repeat-like | 14 | 0.98 | 5.71E-11 |
| 12 | IPR011989 | Armadillo-like helical | 14 | 0.98 | 5.71E-11 |
| 13 | IPR000866 | Alkyl hydroperoxide reductase/ Thiol specific antioxidant/ Mal allergen | 13 | 0.91 | 9.88E-06 |
| 14 | IPR015943 | WD40/YVTN repeat-like | 13 | 0.91 | 9.88E-06 |
| 15 | IPR000719 | Protein kinase, core | 11 | 0.77 | 1.82E-05 |
| 16 | IPR011990 | Tetratricopeptide-like helical | 11 | 0.77 | 1.82E-05 |
| 17 | IPR014001 | DEAD-like helicase, N-terminal | 10 | 0.7 | 1.08E-07 |
| 18 | IPR003593 | ATPase, AAA+ type, core | 9 | 0.63 | 1.34E-04 |
| 19 | IPR013026 | Tetratricopeptide region | 9 | 0.63 | 3.60E-05 |
| 20 | IPR014021 | Helicase, superfamily 1 and 2, ATP-binding | 9 | 0.63 | 7.66E-08 |
| 21 | IPR002423 | Chaperonin Cpn60/TCP-1 | 8 | 0.56 | 6.81E-06 |
| 22 | IPR002452 | Alpha tubulin | 8 | 0.56 | 5.12E-08 |
| 23 | IPR002453 | Beta tubulin | 8 | 0.56 | 4.25E-07 |
| 24 | IPR008950 | GroEL-like chaperone, ATPase | 8 | 0.56 | 4.25E-07 |
| 25 | IPR011545 | DNA/RNA helicase, DEAD/DEAH box type, N-terminal | 8 | 0.56 | 1.33E-07 |
| 26 | IPR001680 | WD40 repeat | 7 | 0.49 | 6.51E-02 |
| 27 | IPR003578 | Ras small GTPase, Rho type | 7 | 0.49 | 5.64E-04 |
| 28 | IPR003579 | Ras small GTPase, Rab type | 7 | 0.49 | 5.64E-04 |
| 29 | IPR004087 | K Homology | 7 | 0.49 | 3.22E-06 |
| 30 | IPR013785 | Aldolase-type TIM barrel | 7 | 0.49 | 5.71E-02 |
